# Factors Influencing Job Satisfaction and Anticipated Turnover among Nurses in Sidama Zone Public Health Facilities, South Ethiopia

**DOI:** 10.1155/2014/909768

**Published:** 2014-02-24

**Authors:** Agezegn Asegid, Tefera Belachew, Ebrahim Yimam

**Affiliations:** ^1^School of Nursing and Midwifery, College of Medicine and Health Science, Hawassa University, Hawassa, Ethiopia; ^2^Department of Reproductive Health, College of Public Health and Medical Science, Jimma University, Jimma, Ethiopia

## Abstract

*Background*. Workplace turnover is destructive to nursing and patient outcomes as it leads to losing competent and qualified nurses. However, developments of coping strategies demand a clear understanding of workplace variables that either motivate nurses to remain employed or lead them to leave their current jobs*. Objective*. This study was designed toassess factors influencing job satisfaction and intention to turnover among nurses in Sidama zone public health facilities, in Southern Ethiopia. *Method*. Cross-sectional study design was carried out on 278 nurses using both qualitative and quantitative data collection methods from May 12 to June 05, 2010. *Result*. A total of 242 nurses were interviewed giving a response rate of 87%. Nearly two-third (68.6%) of the participants were female, and the mean age was 28 (±6.27) years for both sexes. All job satisfaction subscale except benefit and salary subscale were significant predictors of overall job satisfaction. Satisfactions with work environment and group cohesion (AOR: 0.25 [95% CI: 0.12, 0.51]), single cohesion (AOR: 2.56 [95% CI: 1.27, 5.13]), and working in hospital (AOR: 2.19 [95% CI: 1.12, 4.30]) were the final significant predictors of anticipated turnover of Sidama zone nurses. *Conclusions*. More than any factors managers should consider the modification of working environment and group cohesions rather than trying to modify nurses to retain and maintain more experienced nurses for the organizations.

## 1. Introduction

### 1.1. Background

Nurse turnover is a costly problem that will continue as healthcare faces the impending nursing shortage; a new generation of nurses enters the workforce, and incentives provided to nurses to work for institutions increase. A variety of factors influence the retention of nurses in adult care settings, including work satisfaction, group cohesion, job stress, and work schedule [1]. Again, recruitment, retention, turnover, and development of quality care in nursing are global issues within the health care setting [2].

Although the definition of turnover varies according to different literature, employee turnover is defined as the ratio of the number of workers that had to be replaced in a given time period to the average number of workers [3], and generally viewed as the movement of staff out of an organization [4].

Employee job satisfaction is the fulfillment, gratification, and enjoyment that comes from work. It is not just the money or the fringe benefits, but the feelings employees receive from the work itself. The most used research definition of job satisfaction is by Locke who defined it as “*a pleasurable or positive emotional state resulting from the appraisal of one's job or job experiences.*” Implicit in Locke's definition is the importance of both effect, or feeling, and cognition, or thinking. When we think, we have feelings about what we think [5]. In another way, it is defined simply as how people feel about their jobs and different aspects of their jobs. It is the extent to which people like (satisfaction) or dislike (dissatisfaction) their jobs [6]. Wikipedia also defines job satisfaction as a pleasurable emotional state resulting from the appraisal of one's job, an affective reaction to one's job, and an attitude towards one's job [7].

The ability to produce, the quality of the work, the opportunity to learn and express creativity, the sense of pride in their profession, the recognition for a job well done, the ability to work well in a team, the social satisfaction derived from relationships at work, the opportunity to experience personal growth and the rewards from a physically supportive work environment, and autonomy are all factors that impact job satisfaction [1, 8].

Work satisfaction is comprised of intrinsic and extrinsic factors. Intrinsic factors are those internally derived and include personal achievement, sense of accomplishment, and prestige. Extrinsic factors are those derived from factors in the practice environment and include pay and benefits, working conditions, and resources [1].

To study job satisfaction many theorists have been identified. Edwin A. Locke's range of affect theory [9] is arguably the most famous job satisfaction model. The main premise of this theory is that satisfaction is determined by a discrepancy between what one wants in a job and what one has in a job. Further, the theory states that how much one values a given facet of work (e.g., the degree of autonomy in a position) moderates how satisfied/dissatisfied one becomes when expectations are/are not met. When a person values a particular facet of a job, his satisfaction is more greatly impacted both positively (when expectations are met) and negatively (when expectations are not met), compared to one who does not value that facet [7].

Another well-known job satisfaction theory is the dispositional theory. It is a very general theory that suggests that people have innate dispositions that cause them to have tendencies toward a certain level of satisfaction, regardless of one's job. This approach became a notable explanation of job satisfaction in light of evidence that job satisfaction tends to be stable over time and across careers and jobs. Research also indicates that identical twins have similar levels of job satisfaction [7].

Frederick Herzberg's two-factor theory (a.k.a. motivator hygiene theory) attempts to explain satisfaction and motivation in the workplace. This theory states that satisfaction and dissatisfaction are driven by different factors—motivation and hygiene factors, respectively. An employee's motivation to work is continually related to job satisfaction of a subordinate. Motivation can be seen as an inner force that drives individuals to attain personal and organization goals. Motivating factors are those aspects of the job that make people want to perform, and provide people with satisfaction, for example, achievement in work, recognition, and promotion opportunities [7].

Hackman and Oldham proposed the job characteristics model, which is widely used as a framework to study how particular job characteristics impact job outcomes, including job satisfaction. The model states that there are five core job characteristics (skill variety, task identity, task significance, autonomy, and feedback) which impact three critical psychological states (experienced meaningfulness, experienced responsibility for outcomes, and knowledge of the actual results), in turn influencing work outcomes (job satisfaction, absenteeism, work motivation, etc.). Job satisfaction describes how content an individual is with his or her job. The happier people are within their job, the more satisfied they are said to be. Job satisfaction is not the same as motivation, although it is clearly linked. The most common way of measurement of job satisfaction is the use of rating scales where employees report their reactions to their jobs. Questions relate to rate of pay, work responsibilities, variety of tasks, promotional opportunities in the work itself, and coworkers [7].

### 1.2. Statement of the Problem

All nursing associations and unions report a deteriorating quality of work-life for nurses. Quality of work-life is widely believed to be one of the most important factors in recruitment and retention, thus having an impact on the current and the future supply of nurses. To deal with the problem, the range of issues includes appropriate workload; professional leadership and clinical support, adequate continuous professional education, career mobility and career ladders, flexibility scheduling and deployment, professional respect, protection against injuries and diseases related to the work place, and good wages [10].

Peru stated that a major challenge for national, regional, and local governments is the implementation of strategies to reduce maternal mortality and chronic child malnutrition so as to create conditions for sustainable development. Achieving these results requires not only adequate implementation of health services but also “placing the right people in the right places, obtaining a fair distribution of health professionals in different regions, according to the different health needs of the population” (Toronto Call to Action Towards a Decade of Human Resources in Health for the Americas, 2006–2015). This means confronting common problems in the management and development of human resources, including limited development of health personnel competencies, health personnel in remote areas who lack access to training opportunities, poor coordination with training institutions whose training does not meet regional needs, training programs carried out in settings different from the actual work context, no performance evaluation based on competencies, and high turnover rates for trained staff [11].

Job satisfaction's consequence can be discussed from different points of views; Maslow [12] suggests that employees will always tend to want more from their employers. When they have satisfied their subsistence needs, they strive to fulfill security needs. When jobs are secure they will seek ways of satisfying social needs and if successful will seek the means to the ultimate end of self-actualization. The most important correlate of work satisfaction is retention. Employees who are satisfied with their work tend to remain in their jobs [5].

Intuitively, it is easy to link patient safety and patient satisfaction to employee satisfaction. A happy employee is focused on their professional tasks, without being distracted by a negative environment, which leads to better performance. Statistically valid research is turning this intuition into fact. For example, the Georgia Quality Initiative, which began in 2003, has identified that long term care (LTC) facilities with happy employees have fewer patient falls, and fewer residents with acquired pressure ulcers and acquired catheters. There is also statistically valid evidence that shows a strong relationship between patient satisfaction and employee satisfaction [13].

Nurses who were not satisfied at work were also found to distance themselves from their patients and their nursing chores, resulting in suboptimal quality of care. Dissatisfaction with work can cause poor job performance, lower productivity, and staff turnover and is costly to organizations. The relationship between job satisfaction and performance was found to be even higher for complex (e.g., professional) jobs than for less complex jobs [5]. In addition, most importantly there is growing evidence of the association between health care workers' job satisfaction and the outcome of health care. Stress and illness contribute to poor clinical judgment, risking harm to patients; stressed workers are vulnerable to injury and have a higher absenteeism rate [14].

Although some turnover can revitalize an organization, a high voluntary turnover rate can have negative consequences. These include costs associated with recruitment and orientation of new nurses, loss of experienced nurses, periods of short staffing accompanied by overtime for remaining RNs, or use of temporary agency staff who are less familiar with the setting than employees, and potential for increase in adverse patient outcomes [15].

Career opportunities and training afford individuals the prospect of further developing themselves and growing within the ranks of their career. They also acknowledge experience and time dedicated to nursing, which provides much-needed recognition in the field of nursing. Low wages, lack of resources to work effectively, limited career opportunities, and limited educational opportunities are other important factors. Because of insufficient staffing levels nurses become frustrated with their inability to complete their work to their professional satisfaction, and they experience difficulties in meeting patient's needs [4].

Once the comparison of the “gap” between capabilities needed in the organization and those existing in employees is identified, then training and development activities must be designed. The focus throughout this is providing guidance to employees and creating awareness of career growth possibilities within the organization. For many individuals, continuing to enhance their capabilities and knowing that there are growth opportunities in the organization may lead to greater job satisfaction and longer employment with that organization [16].

The relationships of nurses with physicians may be very positive, but it also results in some of the most stressful encounters for nurses. Because this stress plays a key role in job satisfaction, a number of studies have been done focusing on nurse/physician relationships. When these relationships lack respect and trust, the result is a difficult working environment and potential for ineffective patient care. Interpersonal relationships always are the result of actions by both parties, and both are responsible for their success [17]. As supplementary to this the result of the survey of more than 10,000 nurses in Texas shows 82% of staff nurse and 77% of nursing directors had experienced verbal abuse in their practice and verbal abuse was responsible for 16–18% of the turnover rate of staff nurse and 18–42% of the turnover rate for directors of nursing.

According to Herzberg two-factor theory of job satisfaction, people are made dissatisfied by a bad environment, but they are seldom made satisfied by a good environment. The prevention of dissatisfaction is just as important as encouragement of motivator satisfaction [18]. Investigating possible changes over time in sources of dissatisfaction revealed that factors related to the work environment rather than individual or demographic factors were still of most importance to nurses' turnover intentions. The differences found to occur across work settings necessitates analysis of job satisfaction at ward level, and the contribution of qualitative methods to develop more insight that is detailed is emphasized. Predictors of registered nurse (RN) turnover have included intent to leave, nurse job dissatisfaction, salary, fewer years on the job, not enough time to do the job well, and demographic characteristics (male, unmarried, nonwhite, and no children living at home are more likely to leave) [15]. According to Rambur et al. intent to leave for reasons associated with job dissatisfaction was higher than leaving for reasons associated with career advancement or situational reasons across all educational preparations [19].

In addition to improved patient satisfaction, other benefits of measuring and improving employee satisfaction include reduced turnover, associated reductions in training costs, identifying cost-saving opportunities, curbing absenteeism, strengthening supervision, evaluating patient-service issues, assessing training needs, streamlining communication, benchmarking the facility's progress in relation to the industry, and gauging employee's understanding of, and agreement with, the facility's mission [1].

Today's global nursing shortage is having an adverse impact on health systems around the world. A major initiative by the International Council of Nurses (ICN) yielded important information regarding the shortage and solutions to it. These are organized into five priority areas: policy intervention; macroeconomics and health sector funding; workforce planning and policy, including regulation; positive practice environments; retention and recruitment (includes migration); and nursing leadership. International momentum is building, providing the opportunity to bring attention to these issues and to take action [20].

The rapid expansion of training and health service institutions creates a major gap in terms of human resource for health (HRH) as trainers, service providers, and managers. There have been a lot of efforts by the government to bridge this gap. Moreover, the health policy of Ethiopia emphasizes training of community based task-oriented frontline and midlevel health workers. As a mechanism to retain health workers, the policy supports developing an attractive career structure; remuneration and incentives for all categories of workers within their respective systems of employment have been considered [21].

The 2006 WHO estimates indicate that Ethiopia has one of the lowest health workers per population ratio of the 57 crisis countries with one health worker for every 4,050 people, and the highest estimated shortage of 152,040 health workers needed to reach the target 2.3/1000 ratio. To deal with this the G8 endorsement of the Agenda for Global Action (AGA) has been mapped six recommendations against the problem based on study done on four Sub-Saharan countries. From that, AGA 3 and 4 are about scaling up health worker education and training, retaining an effective, responsive, and equitably distributed health workforce [22].

## 2. Literature Review

### 2.1. Literature Review

The nurse workforce in Sub-Saharan Africa (SSA) is a significant component of its health workforce, perhaps more than on other continents. Nurses constitute 45–60 percent of the entire health workforce with nurses responsible for a broad range of services. Generally, the nurse to physician ratio is much higher in SSA than on other continents which is between 20 : 1 and 11.6 : 1 in Tanzania and Ghana, respectively, to a low ratio of 2 : 1 and 2.5 : 1 in Madagascar and Central Africa Republic. While the ratio of nurses to doctors may be high, the ratio of nurses to population in SSA tends to be much lower than in most other regions of the world [23].

In the study undertaken in United State at different times, the national turnover rate for hospital nurses was 12% in 1996, 15% in 1999, and 26.2% in 2000. In the study done specifically in north central West Virginia, job satisfaction significantly correlated with context, structure, and attitudes variables even though age was not correlated with job satisfaction [1].

In a study conducted in Kuwait to assess nurse job satisfaction they found significant relationship of job satisfaction with marital status with positive. However, a higher level of education qualification showed an invasive relationship with job satisfaction [24] which is also supported by Larrabee et al. [15]. But other nurse researchers indicated younger nurses with less work experience, lower professional titles, and lower working positions experienced lower levels of emotional exhaustion which is positively related to job satisfaction and negatively to intention to turnover [25]. Again this idea contradicts Cameron et al.'s who was said RNs with more years of experience had highest job satisfaction, lowest burnout, and were less likely to leave [26, 27].

Age of worker was also considered as one influencing factor of job satisfaction and turnover. In a study done in china, for 20–30-year-old nurses, work satisfaction and job stress were significant predictors of anticipated turnover. For the 31–40-year-old nurses, work satisfaction was predictive of anticipated turnover. For the 41–50-year-old nurses, work satisfaction and group cohesion was predictive of anticipated turnover. For the nurse who is 51 or older, there were no significant predictors of turnover [1, 25]. In Canada turnover of nurse in relation to years of service was that 30% of new nurses left their jobs in the first year and 57% resigned in their second year [28]. For American nurses, overall nursing experience was significantly related to intention to leave (*F*_(9,38)_ = 2.64,  *P* = 0.02). Nurses with less than 1 year experience and between 10 and 11.5 years experience were significantly less likely to indicate intention to leave than those with 1–1.5 years experience (*P* values < 0.05). The reduced form specification was similar to the instrumental variable model results [29].

Nursing is one of numerous professions in which one sex comprises the clear majority of workers while gender difference reported as factor for job satisfaction and nurse turnover. In the study conducted in Maryland to investigate reason of leaving work among women and men they found 69.7% of men cited better salaries as a reason for leaving nursing in contrast to only 32.6% of women and 63.6% of men found their current position more rewarding professionally compared with only 46.1% of women [30]. It is not only the reason that differ, but also their rates of leaving reported on the surveys of nurses have shown significant differences in rates of leaving nursing between men and women; 7.5% of men nurses dropped out of nursing within 4 years of graduating from nursing school compared with 4% of women [31].

Szigeti et al. investigate factors relating to RNs' and LPNs' desire to keep practicing in rural hospitals; they found that overall job satisfaction and performance constraints are a predictor of turnover intent for RNs and LPNs. Satisfaction with promotion is the only work related predictor of turnover intent for RNs [32] and Davidson et al. also agreed that intent to leave predicted by perception of little promotion, low decision latitude and poor communication [33]. Performance constraints, role ambiguity, and shift work were the only work related turnover predictors for LPNs [32].

Promotion is one of the motivating factors which can effectively prevent dissatisfaction of workers [18] but in a study conducted on 500 (87.2% response rate) Kuwaiti nurses, nurses were found to be dissatisfied with professional opportunities and extrinsic rewards while they were satisfied with praise and recognition, scheduling of the duty, and controls and responsibility [24]. Another study done on Palestinian nurses shows lack of career advancement opportunities and unsupportive hospital policies and practices contributed to job dissatisfaction [34]. As Saari and Judge indicate on their result of multigenerational analysis of nursing working force, they show providing and supporting education or career-development opportunities may be another strategy to increase overall job satisfaction for nurses [5].

The more job stress, the lower group cohesion and the lower work satisfaction, the higher the anticipated turnover. Again the higher the work satisfaction, the higher group cohesion and the lower anticipated turnover. The more stable the schedule, the less work-related stress, the lower the anticipated turnover, the higher group cohesion and the higher work satisfaction [1].

A cross-sectional survey of 664 registered nurses (RN) on 34 acute care in-patient hospital wards was done in 2008 on a Finnish university hospital to assess nurses' work environment and nursing outcomes and it indicate that only 7% of the respondents answered that they did not feel any job-related stress. More than one-fifth (21%) of the respondents felt stress quite a lot. Those who felt that respect and relationships were lower also felt job-related stress rather much or exceedingly [35].

As indicated in many study motivation guide actions of person but on survey of 147 South African nurses indicated serious morale problems. Over 40 percent of the respondents agreed with the statement that they “dreaded” the next day at work, felt unmotivated (over 50%), could imagine working overseas (50%), and intended to leave their jobs (40%). Unhappiness with their vocational choices, stress at work, and difficulty with change were significantly related to burnout and demotivation [36].

Overall, professional nurses in South Africa were marginally dissatisfied (mean 2.94). They did, however, express greatest satisfaction in their relationship with patients and the gratification they obtained from patient care (3.734), their relationship with their nursing colleagues (3.58), doctors (3.391), and their sense of belonging in the communities within which they work (3.37). They were most dissatisfied with their pay (2.02), the workload (2.24), their career development opportunities (2.59), and the resources available to them (2.73) [37].

In the same study (South Africa), female nurses were generally more satisfied with resources than their male colleagues but no statistically significant and overall gender differences in intention to leave were found in the study of Rambur et al. [19]. While nurses above 40 were significantly more satisfied than their younger colleagues with their relationships with management and doctors, nurses with more than 20 years' experience were also significantly more satisfied than their less-experienced colleagues with most of the facets of their work [37]. That is why Kiyak et al. conclude that intent to leave predicted by age (younger), length of employment (shorter), job dissatisfaction, and type of agency work (community) [38]. Specifically nurses aged 51 or over are less likely to leave for reason of job dissatisfaction compared to nurses in the 40–50 age groups [19].

According to the European Commission in its review on the progress on quality in work in 2003, “despite the strong employment performance observed in European labour markets in the second half of the 1990s, recent data on the evolution of job satisfaction and job quality … over this period do not indicate significant changes in quality in work. Only in Greece and Portugal there was a significant decrease in the share of employees expressing low satisfaction with their type of work. On the other hand, job satisfaction seems to have deteriorated somewhat in Italy in the 1996–2000 periods. In 2000, in the EU overall, around 20% of all employees still declared themselves dissatisfied with their job. Relatively high degrees of dissatisfaction in Greece, Italy and Spain contrast with very high shares (90% or more) of employees who are satisfied with their job in Denmark, France, Ireland, the Netherlands and, most notably, Austria” [39].

Many studies report a consistent and negative relationship between job satisfaction and turnover, as dissatisfied employees are more likely to leave an organization than satisfied ones [31] and they found the major predictor for intention to leave a job is dissatisfaction and the major predictor of job satisfaction is psychological empowerment [15].

In general a survey of hospital health worker in Uganda showed that the important correlates of intention to stay or job satisfaction include the importance of salary (but not the satisfaction with salary, which is uniformly low), a good match between the job and the worker, active involvement in the facility, a manageable workload, supportive supervision, flexibility to manage the demands of work and home, job security, and a job perceived as stimulating or fun [40].

In the metaanalysis of 13 studies in Taiwan, they list ten factors related to experienced turnover: poor promotion opportunity, work stress due to high workload, lack of continuing education, dissatisfaction with salary, superior, inflexible scheduling, administrative policies, recognition, unstable scheduling, and dissatisfaction with fringe benefits [19].

Even though some developed countries reports indicate about 20% turnover; among Uganda nurses, study indicates interest in leaving Uganda or the health profession (80% intended to stay in their jobs at least three years), and with 85% still in their first jobs specifically older workers in general are more satisfied than their younger. Again, they found the average overall health worker job satisfaction to be neutral—about 3.2 on a scale of 1 to 5. The average, however, masks a bi-modal finding—health workers were either satisfied (48.7%) or dissatisfied (35.3%), with only 16% reporting that they were “neutral” [40].

The correlation between the level of intention to stay at work and recognition for competent/satisfactory performance of nurses was positive but insignificant (*r* = 0.11, *P* = 0.06), whereas the correlations between intention to stay at work and recognition for outstanding/excellent performance and recognition for achievements were (*r* = 0.21, *P* < 0.01) and (*r* = 0.23, *P* < 0.01), respectively. That is, nurses who perceived having more recognition for their outstanding performance or their achievements reported a higher level of intention to stay at work than nurses with less recognition. Likewise, the correlation between job stress and the level of intention to stay was significant (*r* = 0.21, *P* < 0.001) [41].

In the study done in Belgium to investigate interrelationships between the nurse practice environment and burnout, Poor organizational environments at different levels appear to lead to feelings of exhaustion, cynicism, and inefficacy, which in turn reduce job satisfaction, increase risks of departure from the organization or nursing practice, and have potentially negative impacts on quality of care [42]. Again in study, 1,521 registered female nurses working in hospitals were under one-year follow-up analyses by multivariate Poisson regression. In china the conclusion was that unfavorable psychosocial work environment predicts intention to leave in Chinese nurses [43]. As Saari and Judge indicate, when employees are asked to evaluate different facets of their job such as supervision, pay, promotion opportunities, and coworkers, the nature of the work itself generally emerges as the most important job facet [5].

According to the survey data from Spain, Finland, and the Czech Republic, job satisfaction seems to increase with worker participation or involvement in the organization where they work. Data from the Spanish SQLW 2004 clearly show that the more workers are able to participate in working decisions, the more satisfied they are with their job. Similarly, data from the Finnish QWLS 2003 also indicate that workers who are “at least sometimes able to take part in the planning of own work” or “able to apply own ideas in work” are significantly more satisfied or less dissatisfied than those who are “never” able to contribute to their work in this way [39]. On the other hand Sourdif indicates that work satisfaction and satisfaction with administration are the most significant predictors of intent to remain and explained 25.5% of variance in intent to remain [28].

In a study conducted on 209 general hospital nurses of Hong Kong with response rate of 50% they found insignificant relation between autonomy and job satisfaction [44]. According to Nguyen et al., one of the variables that may be expected to influence job satisfaction is “the degree of perceived autonomy that workers enjoy in the way they do their job.” The expected relationship is that more autonomy is associated with greater job satisfaction. The Danish DWECS survey provides an interesting finding regarding the relationship between job satisfaction and job autonomy. According to its results as job decision latitude decreases, fewer respondents report a high degree of job satisfaction. Almost 90% of male employees and almost 85% of female employees with high job decision latitude are satisfied to a high degree, while only about 56% of those with low job decision latitude report a high degree of job satisfaction [39].

### 2.2. Conceptual Framework

This conceptual framework is adapted to our context and research interest from the work of Irvine and Evans and the literature review. Initially they presented a model based on Mueller and Price's theory that different disciplinary perspectives contribute to explaining nurse turnover: economists who emphasize individual choice and labor market variables, sociologists, who emphasize the structural characteristics of the work environment and work content, and psychologists, who emphasize individual variables and intrapsychic processes [45].

### 2.3. Significance of the Study

After a review of the literature specific to nurse job satisfaction and intention to turnover we just realize the importance of the issue in our country and more than that many researches were undertaken by developed countries but little were found in developing countries specifically Ethiopia. The lack of research addressing the factors that influence nurses' job satisfaction and intention to turnover is a problem because if nurse administrators do not know what the nurses want, they cannot make changes to better satisfy the nurses. This study provided information that is important to practice, nursing administration and policy maker, and nursing education.

*For Policy Maker.* This study will provide input for policy maker on changing worker characteristics, changing job characteristics, and working environment adjustment. It may also help in job placement strategies to retain more staff. Again, for education and training of staff this study may indicate the area of interest for provision of problem solving education. 

*For Health Manager and Patient Outcome.* Administrators could use this information to build solid and supportive units. This is important because the culture of the unit and the quality of nursing staff affects every aspect of a nurse's practice and the patients' care. Health care managers and practitioners should be aware of the hardships that nurses face in trying to give quality care to patients. Therefore, it can be said that knowing a nurse's tendency and difficulties will help predict the nurse's job satisfaction and intention to quit. High intention to quit and low job satisfaction might lead to a decrease in service quality and patients' intention to return for future use and an increase in the costs for patient care. However, determining how to best capture and quantify nurse turnover costs and its consequence can be challenging because it costs to understand the most determinant factors causing those turnover.

No one ever determined the factors affecting nursing staff's job satisfaction and intention for turnover and its impact on the institution, patient, and generally on the nursing system. That is why this study aims at the amplification of the magnitude and association of job satisfaction with intention for turnover among nurse of Sidama zone, which helps more powerfully the health manager to know the most influential factors to influence staffs and maintain workers.

## 3. Objective

### 3.1. General Objective

To assess factors influencing job satisfaction and intention to turnover among nurses in Sidama zone public health facilities Southern Ethiopia.

### 3.2. Specific Objective

1. To determine the level of nurses' overall job satisfaction in Sidama zone public health facilities,
2. To identify factors influencing job satisfaction of nurses in Sidama Zone Public Health Facilities,
3. To identify factors influencing intention to turnover among nurse in Sidama Zone Public Health Facilities,
4. To identify factors of job satisfaction that best predict intention to turnover nurse working in Sidama Zone Public Health Facilities.

## 4. Methods and Material

### 4.1. Study Area and Period

The study was carried out from May 12 to June 05, 2010, in Sidama zone SNNPR Ethiopia. Sidama is bordered in the south by the Oromia Region except for a short stretch in the middle where it shares a border with Gedeo, in the west by the Bilate River which separates it from Semien Omo, and in the north and east by the Oromia Region. The administrative center for Sidama is Awasa; other towns include Yirgalem and Wando. Hawassa is located at the eastern shore of Lake Awassa 275 km south of Addis Ababa. Sidama zone has a population of 3,232,306 people in 19 woreda with three city administrations. There are around 994 health care professionals, 75% (750) of them are nurses working in governmental hospitals and health centers in the zone.

### 4.2. Study Design

Cross-sectional study design with qualitative and quantitative methods was implemented to assess the factors influencing job satisfaction and anticipated turnover among nurses of Sidama Zone public health facilities Ethiopia.

### 4.3. Populations

#### 4.3.1. Source Population

All nurse hold diploma and are above qualified and have 6 months and greater work experience, who were working in public hospitals and health centers.

#### 4.3.2. Study Population

All randomly selected nurses working as full timers in selected hospital and health center in Sidama zone having a 6-month and greater work experience participated in the study.

Head nurses of hospital wards, health center heads, and woreda health department officers were key informants for the in-depth interview.

### 4.4. Inclusion and Exclusion Criteria

#### 4.4.1. Inclusion

All nurses having diploma and above qualification were involved in the study.

#### 4.4.2. Exclusion

All nurses who had a work experience of less than 6 months at the time of the study were excluded.

### 4.5. Sampling

#### 4.5.1. Sample Size

Consider

(1) $$\begin{array}{c} n=\frac{{\left(Z_{\alpha /2}\right)}^{2}P\left(1-P\right)}{d^{2}}, \end{array}$$

*n*—minimum sample size, *P*—estimated proportion of nurses job satisfaction (50%), *d*—the margin of sampling error tolerated (5%), *Z*_*α*/2_—the standard normal variable at 1 − *α* % confidence level (5% = 1.96)

(2) $$\begin{array}{c} n=\frac{{\left(1.96\right)}^{2}0.5\left(1-0.5\right)}{{\left(0.05\right)}^{2}}=384. \end{array}$$

Since the sample size is greater than 5% of the total population, finite population correction formula was used.

- Correction for finite population < 10,000 = nf (final sample size) = *no*/(1 + (*no*/*N*)) = 252
- By adding 10% nonresponse rate, the used final sample size for quantitative study was 278 individual nurses from different hospitals and health centers in the zone.
- Seven in-depth interviews were conducted in which 2 of them were from health center head, 2 woreda health department head, and the rest 3 were from unit head nurse in the hospital. Here with current reform (BPR) there is no position for matron; instead all health care personnel were categorized into different case teams and discussed all positive and negative aspects of their work there, but if the problem needs greater attention and concern they may report directly to the medical director.

#### 4.5.2. Sampling Technique

To select 278 nurses from 34 health center and three governmental hospitals simple random sampling and proportional allocation to size were employed.

*For Health Center. *Simple random sampling technique was used to select health center from each woreda after proportionally allocating a number of actual nurses who contributed from each woreda in the zone. Accordingly, 16 health centers were selected from 19 different woredas in the zone by considering 85% coverage. All nurses working in selected institutions during data collection and those who fulfill the inclusion criteria were involved in the study. Additionally, one health center was included from each city administration (Hawassa, Aleta wondo, and Yirgalem).

*For Hospitals*

- *Step one*: all hospital were listed down with their respective nurse number, and then the proportion that they contribute for the sample was calculated.
- *Step two*: proportion changed to countable nurse number.
- *Step three*: simple random sampling method was employed to have the desired number from each hospital.

Purposively the PI invite two health center heads, two woreda health department head, and three head nurses from hospital to involve them in the in-depth interview. Saturation and redundancy of ideas were used to limit the questions and number of in-depth interviews to eight.

### 4.6. Variables

#### 4.6.1. Dependent Variables

- Intention/anticipation to turnover.
- Job satisfaction.

#### 4.6.2. Independent Variable

- Sociodemographic data (age, sex, work experience, marital status, unit, working institution, and educational status).
- Benefit and salary.
- Autonomy.
- Professional training.
- Recognition of nurses performance at work.
- Leadership relationship.
- Promotion.
- Working environment and group cohesion.

### 4.7. Operational Definition and Measurement

*Nurse Turnover*. Ratio of registered nurses (RNs) who leave in 1 year to the number of budgeted full-time equivalent RN positions includes involuntary and voluntary employment termination.

*Overall Nurse Job Satisfaction (Table 6).* Nurse job satisfaction is positive or pleasurable emotional state resulting from the appraisal of one's job or job experience. Job satisfaction of nurse by determinant factors was measured using the questionnaire adopted and adapted by principal investigator with a 32-item scale. This instrument has 5-point Likert scale in which 5 denotes very satisfied and 1 denotes very dissatisfied. When the total score for job satisfaction subscale is greater than computed mean, we say they were satisfied on overall aspect of their work. Based on value of computed mean of mean that was computed from individual response of study participant.

*Job Dissatisfaction.* It is a feeling of unhappiness about the work that one does in his/her own appraisal of work. 

*Intention/Anticipation to Leave a Job (Table 7)*. The intent or predisposition to leave the organization where one is presently employed or employee's plan of intention to quit the present job and look forward to find another job in the near future. It was measured using two items with assessment of attitudes on current job and reason for leaving organization for workmate in the past. The question scored from three-point Likert scales in which 1 denotes agreement and 3 denotes disagreement. When individual study participants scored below overall mean, they were considered as having intention to leave the institution. 

*Autonomy.* Job characteristics that enable nurses to make individual decisions about daily practice and also it feeling of nurse about independence in the work. Autonomy was measured by using four items with five-point Likert scale in which 1 denotes very dissatisfied and 5 denotes very satisfied. Respondents considered autonomous and satisfied about it when they score above computed mean for the subscale. 

*Work Environment and Group Cohesion*. It is operationalized as professional practice and that is perceptions of integration into the organizational and colleague environment. This was measured using the nine-item work environment and group cohesion scale in which 1 denotes very dissatisfied and 5 denotes very satisfied. Consider satisfaction level of this subscale as satisfied with environment and cohesion when respondents scored above their computed mean. 

*Promotion*. It is the perceived interest (satisfaction) in one's career development in the organizations. This aspect of job satisfaction was measured by using three items with 5-point Likert scale with one denoting very dissatisfied and 5 denoting very satisfied. Consider satisfaction level on promotion when respondent were scored above their computed mean. 

*Perceived Alternative Employment Opportunity*. It is the Perceived employment opportunity outside the organization that potential individual can join. This was measured by using three items with 5-point Likert scale with 1 denoting very dissatisfied and 5 denoting very satisfied. Consider satisfaction level on perceived alternative employment opportunity when respondents scored above their computed mean. 

*Recognition at Work*. It is the feeling of being valued by the organization administration. This was measured by using four items each scored in 5-point Likert scale with 1 denoting very dissatisfied and 5 denoting very satisfied. We consider that staff nurse were satisfied by the level of recognition in the organization when they scored above their computed mean of their individual mean. 

*Benefit and Salary*. It is the level of satisfaction with wage in the organization and it was measured by using three items each scored with five-point Likert scale with 1 denoting very dissatisfied and 5 denoting very satisfied. We consider that staff nurses were satisfied by the level of benefit and salary in the organization when they scored above their computed mean of their individual mean. 

### 4.8. Data Collection and Instruments

#### 4.8.1. Instruments

Quantitative data were collected using a structured questionnaire that was adapted from the Minnesota Satisfaction Questionnaire (MSQ) short form and Mineser nurse practitioner satisfaction scale to our context. The questionnaire was initially prepared in English and translated to Amharic. Three different individuals who were health professionals checked consistency after back translation to English.

The questionnaire has three major parts: the first was demographic which consists of age, sex, work experience, marital status, working unit, working institution (hospital or health center), and educational status, and the second part was job satisfaction scale. Under job satisfaction scale, there were a total of 32 questions with 8 subscales named as leadership relationship subscale, promotion subscale, autonomy subscale, working environment and group cohesion subscale, professional training subscale, benefit and salary subscale, recognition at work subscale, and perceived alternative employment opportunities subscale. All questions of job satisfaction were rated from 5-point Likert scale with 5 denoting very satisfied and 1 denoting very dissatisfied. The third was turnover intention, which consists of six questions formulated by principal investigator. For this instrument the validity and reliability were tested with pretest prior to main data collection time to Jimma hospital nurse and Jimma higher two-health center. For qualitative part principal investigator formulated eight broad questions that can enables to elicit information regarding research objective and used as guide in interview.

#### 4.8.2. Data Collection Technique

*For Quantitative Part*. The sample population was invited to participate voluntarily by explaining the rationality of the study at the time of data collection. Then structured and pretested self-administered questionnaire was administered to respondent by trained data collector. The questionnaire was distributed by trained data collector to staff nurses in the ward and working unit while they are on the work. Written guideline was given to administrator of the questionnaire to assure that each nurse receives the same directions and information. Anonymity of the participant was kept by informing them not to write their name.

*For Qualitative Part*. The principal instigator controlled and performed the job. Semistructured questionnaire was used to guide in-depth interview. Written informed consent was obtained from staffs then by arranging comfortable time for informants 15- to 20-minute in-depth interview was employed for each key informant (head nurse from health centers and hospital unit and woreda health department head). The interview was recorded and transcribed from audio manually.

Ten diploma nurses were recruited for data collection in health center and three BSc nurses for hospital. In addition, one BSc nurse was recruited for monitoring and supervision of data collection on the spot. For in-depth interview, the principal investigator takes all the responsibility. Before actual date of data collection all data collectors were trained at Hawassa for one day for the objectives of the study, the contents of the questionnaire, issues related to the confidentiality of the responses, and the rights of respondents.

### 4.9. Quality Control

The questionnaire was back translated to English to see the consistency. The instrument was pretested on seven nurses of Jimma specialized referral hospital and seven Jimma higher two-health center nurses (5% of sample). After pretest, certain words and sentences were rearranged. By using SPSS for windows version 16 Crombach's alpha was calculated to test internal consistency (reliability) of items and except leadership subscale most of the tools scores > 0.8. Crombach's alphas were >0.7. However, the result was not incorporated to the main research result.

All data collectors were trained for one day. During training all data collectors were communicated overviews regarding nurse job satisfaction, its impact on health care delivery system, about effective data collection methods, and ethical issue during data collection.

The PI and two recruited supervisors were responsible for supportive supervision on the spot and checking questionnaire on daily basis.

### 4.10. Data Processing and Analysis

Editing and sorting of the questionnaires were done to determine the completeness manually everyday. Data entry and analysis were performed by using SPSS for windows version 16. The responses in the completed questionnaire were coded and entered into a data entry template. Summary tables, figures, and charts were used for describing data. Bivariate analyses between dependent and independent variables were performed using chi-square (*χ* ^2^) and binary logistic regression. The correlations between the variables measured on an interval scale analyzed computing the product-moment correlation coefficients (i.e., Pearson's *r*) (Table 9) which showed the intensity and direction of the relationships between the variables. In this study, ±1.0-0.5 considered strong, ±0.5–0.4 moderate, and ±0.4–0.2 a weak correlation [46]. Hierarchical regression analyses were done to formulate models that best predict nurse job satisfaction and intention to leave the organization. Statistical tests were performed at the level of significance of 5% (Table 8).

The tape-recorded qualitative data were transcribed and translated to English under selected themes based on the question guides and summarized manually. The result was presented in narratives triangulated with the quantitative results.

### 4.11. Ethical Considerations

Ethical approvals for the study were obtained from Ethical Review Committee (ERC) of the College of Public Health and Medical Sciences, Jimma University and Hawassa University. Official letters that were obtained from nursing department were delivered to Sidama Zonal health department and Hawassa referral hospital. The letters of approval and cooperation obtained from the zonal health bureau were presented to all woreda health departments then to the selected health institutions. Informed consent was taken orally from each participant before start of data collection (qualitative) and oral consent from quantitative participants. Confidentiality was assured by indicating they are not requested to write their name on the questionnaire and by assuring that their responses will not in any way be linked to them. In addition, they have been told they have the right not to participate and withdraw from the study.

### 4.12. Plan for Communication of Results

The findings will be presented to the Jimma University scientific community in a defense and the result submitted to the department of nursing college of public health and medical sciences. The findings will also be communicated to the local health planners and other relevant stakeholders at national, regional, and zonal levels to enable them to take and apply research recommendations during their planning process. Publications in peer-reviewed, national, or international journals will also be considered.

## 5. Results

### 5.1. Result 

#### 5.1.1. Sociodemographic Characteristics of Study Participants (Table 1)

Out of proposed 278 samples, 242 nurses were involved making the response rate to be 87%. Nearly two-third (68.6%) were females. The mean (±SD) age of the participants was 28.13 (±6.27) years (28.35 ± 6.79 for females and 27.67 ± 4.98 for males). From total study participants 131 (54.1%) were single and 94 (38.4%) have work experience of 2 years to 5 years. The highest reported place of assignment of nurse in both health center and hospitals was OPD 65 (26.9%), pediatrics (13.6%) and MCH (13.2%), orderly. However, most of nurses working at OPD come from health centers. In the study, 20 BSc nurses and 3 BSc midwives were involved while the rest were diploma graduates 219 (90.5%). From those diploma nurses 162 (66.9) were clinical nurse professionals.

#### 5.1.2. The Job Satisfaction Subscales (Table 2)

This study tried to look at the level of overall job satisfaction of staff nurses based on the eight subscales of job satisfaction.

The lowest levels of job satisfaction were reported for benefits and salary (Mean = 1.76) followed by satisfaction level for promotion (*M* = 2.0), and the highest levels of satisfaction were obtained from work environment and group cohesion (*M* = 3.15) followed by autonomy (*M* = 3.02). The internal reliability estimates in this sample (Crombach's alpha) ranged from 0.64 to 0.86.

*(1) Leadership Relationship*. Leadership relationships were not significantly associated with institution of respondent, sex, marital status, and educational qualification. However, strongly significant associations were seen with intention to leave the organization (*P* < 0.05), employment opportunity (*P* < 0.05), working unit (*P* < 0.05), job security and salary (*P* < 0.05), and professional training (*P* < 0.05) and working unit shows significant association with leadership relationship of nurse in their institutions*.*

Out of 242 respondents, 101 (45.5%) were dissatisfied with administration support for their work while 92 (38%) of respondents answered that they were satisfied with this aspect. By the level of recognition given to their work, similar proportions of the nurses were dissatisfied 99 (45.9%) and satisfied 94 (40.1%). Generally, 138 (57.1%) of the study participants were satisfied with leadership relationship in their institutions.

One woreda health bereau head said

*“…Monthly we encourage them to have regular meeting in which all staffs discuss their problem in case team, this make their relation with their head smooth..”*

*(2) Promotion*. None of sociodemographic variable show significant association with level of promotion available in the institutions. The intention to leave the organization was also not significantly associated with level of promotion available in the organizations.

As shown in Table 3, by the level of support for continuing education 166 (68.6), opportunity for professional growth and support for personal growth 174 (74.9%) and development through education and training 143 (59.1%) were reported as dissatisfying. Generally only 100 (41.3%) of nurses were satisfied with the level of promotion available in the facility. Out of dissatisfied 142 nurses 97 (68%) were from health center while 45 (31.7%) were from hospital (Figure 3). 

*(3) Autonomy*. Autonomy subscale was significantly associated with institution of work whether it is hospital or health center (*P* < 0.05) and working unit (*P* < 0.05). Actually more dissatisfied with autonomy nurses were from hospital. However, the level of autonomy was not significantly associated with age, sex, marital status, work experience, educational qualification, and employment opportunity. 

Hundred and six (43.8%) nurses were satisfied about the level of autonomy in nursing care decision at the work, while 106 (43.8%) were dissatisfied. Regarding the level of support available to be fully accountable for already made decision, 102 (42.1%) of them were satisfied and 80 (33.1%) were responding as they were dissatisfied while the rest 60 (24.8%) kept neutral position. Similarly, almost the same proportions were satisfied 96 (39.7%) and dissatisfied 99 (40.9%) with the freedom they have in using their own judgment in the work setup. Generally, most of the workers were dissatisfied on general autonomy level 128 (52.9%) of which 83 (64.8%) were from health center while 45 (35.2%) were from hospital.

In contrary to quantitative result most of informants reported that nurses are autonomous. One health center head from Aleta Wando woreda said

*“…In all case teams where nurses are assigned they did what they like without intrusion of any body and they refer or treat as they like…”***

*(4) Work Environment and Group Cohesion (Table 4)*. From sociodemographic variable only institutions of respondents were significantly associated with working environment and group cohesion subscale (*P* < 0.05); the rest (age, sex, marital status, educational qualification, unit of assignment, and year of experience) were not significantly associated.

The result from the survey demonstrates that 109 (45%) nurses were satisfied with working environment allowing them to make autonomous nursing care decision while 46 (19.0%) of respondents answered neutral and 87 (36%) were reporting dissatisfaction.

For the level of the working environment enabling the feeling accountable for the decision they have already made, 112 (46.3%) were satisfied while 79 (32.6%) of them reported their dissatisfaction. Considerable number 51 (21.1%) is still kept in neutral position regarding satisfaction level of the working environment enabling the feeling accountable for the decision they have already made.

Regarding the level of adjustment of working environment that suits the patient's need in practice 117 (48.3%) of nurses were satisfied, while 48 (19.8) kept neutral state about the issue. One-third of respondents were dissatisfied with the level of working environment encouraging them to make adjustment on work to suit patient's need.

Most of nurses 104 (43.0%) were dissatisfied with the stimulating action of the working environment while only 86 (35.5%) were satisfied with it. As shown from bar graph below, number of hospital nurses was abundant in very dissatisfaction side (Figure 4).

One hundred and five (43.4%) of study participants reported as they have been satisfied with the level of working environment allowing them to expand their scope of practice while comparably around 97 (40.1%) were not satisfied on this aspect of the work.

Regarding environmental provision of high level of clinical competence, 108 (44.6%) of them were satisfied while 90 (37.2%) were reporting they were not satisfied.

Most of staff nurses were satisfied with relationship among staffs in working environment 147 (60.7%) and by the level of staffs influencing one another positively in the working setup 135 (55.8%).

One respondent from hospital said “*…we are too happy about relationship among us we respect one another as staff member, we invite help from one another if any confusion…..I think this is one powerful think we hope to stay in the hospital because no one want to leave love and joy …”*

Additionally the majority of nurses were satisfied with relationship with physician 110 (45.5%) while 81 (33.5%) of respondents answered that they were not satisfied with the relationship.

Generally 132 (54.5%) of study participants were satisfied with working environment and group cohesion subscale while 110 (45.5%) of them were dissatisfied with it.

Qualitatively, most of informants consider the working environment and group cohesion subscale in the direction of intimacy between them. Almost all are happy and reported all staffs are satisfied with the level of relationship, helping one another and socializing among staffs. 

*(5) Professional Training*. Only sex (*P* < 0.05) and institution of work (*P* < 0.05) were significantly associated with professional training subscale; the rest (age, educational status, marital status, working unit and working experience) were not significantly associated.

By the level of training opportunities available in their institutions the majority 130 (53.7%) of nurses were not satisfied while only 62 (25.6%) of nurses repored satisfaction and the rest 50 (20.7) were neither satisfied nor dissatisfied. Again 106 (43.8%) of nurses were dissatisfied with the appropriateness of the training available for enhancing nursing job performance while only one-third 78 (32.3%) of them reported that they were satisfied. In other aspect 121 (50.0%) of nurse were dissatisfied with the level of orientation to new staff coming to the institutions while only 80 (33.1%) of nurses were satisfied with the level of orientation.

Research is too important in clinical patient care so as to have evidence based practice but most of staff nurses were not satisfied with the level of participation on research activity 150 (60.0%) while only 54 (22.3%) were satisfied.

Qualitatively respondents looked in two aspects. First as for short term in-service training one nurse from health center said “*…when one training came to health center we sit for discussion to select appropriate and relevant person for the specified training…in this aspect all staffs are happy…*”

In the second aspect they see with respect to continuous education, one unit head nurse said “*…previously staffs are few in number and round for further education was frequent … now further education become difficult..we worried about that…they said no curriculum designed for those 10* ^*+3*^ *graduated nurse…we do the job without hope that is the most dissatisfying aspect of job regarding promotion available in our hospital…*”

Generally by the professional training subscale 106 (43.8%) of study participants were scoring above the mean (satisfied) while 136 (56.2%) were scoring below the mean (dissatisfied). 

*(6) Benefits and Salary (Figure 6)*. Regarding the benefits and salary subscale with sociodemographic variables only educational qualification shows significant association (*P* < 0.05). Overall satisfactions of respondents were not significantly associated with benefits and pay subscale

As shown in Table 5, more than three-fourth of respondents were dissatisfied with appropriateness of salary composition for their employment 196 (81.0%), while only 26 (10.7%) respondents answered with their satisfaction. Comparably most of respondents were dissatisfied with the level of employee benefits in organization and pay in relation to cost in their living area 180 (74.4%) and 183 (75.6%), respectively.

Interviewer answered supporting the idea for the quantitative data. One health center head said “*… we nurse are not considered well in salary adjustment last time… but other profession fevered during that..we nurses now ignore the level payment and satisfaction we found from it and we consider only satisfaction we get from our job byitself…the payment is totally unfair*”**

*(7) Recognition at Work*. Most of nurses 188 (48.8) were dissatisfied with the level of sense of value given for what they do in their working organizations. Again by the consideration given to their personal need from the organization 50 (20.7%) feel satisfied, 63 (26.0%) kept neutral, and 129 (53.3%) responded they were dissatisfied.

Almost half 126 (52.1%) of respondents were dissatisfied with the level of consideration given to their opinion and suggestion for change in the work setting or practice while only 60 (24.8%) of them were satisfied. Significant number of study participants 103 (42.6%) were satisfied when 90 (37.2%) of nurses responded they were dissatisfied by peer recognition of work.

In qualitative part, informants are happy about recognition level given from the staffs and administrative bodies. Most of respondents answered consistently that they have meeting with staffs in which good performers were recognized and those low performers were given their correction measure after implementation of the new reform BPR. However, one unit head nurse said “*…one nurse was automatically dismissed from the hospital without stepwise disciplinary method made frustration to the staffs …*”**

*(8) Perceived Alternative Employment Opportunities*. Most of the nurses 94 (38.8%) were happy about the easiness of finding another job if they may quit the current one while 87 (36.0%) of them were dissatisfied with the chance. Given their age, educational level, and economical condition, comparably 90 (37.2%) and 92 (38.0%) were satisfied and dissatisfied with the chance attaining suitable job in another organization, respectively.

Regarding the level of easiness to find another alternative job 104 (43.0%) of nurse were dissatisfied while 80 (33.1%) were satisfied and 58 (24.0%) maintained neutral position.

Generally talking, nurses were satisfied with perceived alternative employment opportunity 141 (58.3%), while 101 (41.7%) of nurses responded that they were not satisfied with the opportunity.

Result from in-depth interview also supported the quantitative data. One head nurse said “*…it's not the issue of age and sex, it is the matter of competence, if one have good skill and competence I think he can join everywhere if she/he loses his/her current job… I perceive still nurses have good opportunity to work…*”

Another health center head said “*…since most of health center activity covered by nurses still we want to have more nurse for our institutions if possible and this also holds similar condition for other health center also…there are plenty of job to find…*”

In contrary to this one respondent said “*… I think the opportunity holds two aspects, for those graduated from private and for those from governmental institutions, usually the former kept less chance of attaining job easily…*” 

*(9) Overall Satisfaction (Figure 5).* Out of 242 study participants 115 (47.5%) of them scored below mean which means they are dissatisfied with their overall aspect of job, while the rest 127 (52.5%) scored above satisfaction level.

In in-depth interview one respondent say “*…I think nurse takes satisfaction from their performance or from relation with client apart from other aspect like administration and level of support for education and incentive…and generally they are somehow satisfied…*”

Regarding the level of overall satisfaction in-depth interview participants said that males are more satisfied than females but regarding the level of turnover intention, they ranked as first. One health center head said

*“… As known most of staff professional are females but according to our context I think males are performing well and satisfied more than female on job…”*

Generally, regarding job satisfaction subscale four of them were reported as satisfying aspects of the job by the majority of respondents; these were leadership relationship 138 (57.0%), work environment and group cohesion subscale 132 (54.5%), recognition at work subscale 122 (50.4%), and perceived alternative employment opportunity subscale 141 (58.3%). The rest of four subscales were reported as dissatisfying job aspects.

As shown from Figure 8, the age of respondent increases as the level of satisfaction on job increase. Accordingly, the level of satisfaction for the age group of 20–30 years was 48.7% for 31–49 years 59% and for age group of 41–50 years 10 (83.3%). These results were consistent and supported by qualitative result.

#### 5.1.3. Intention to Leave

Out of 242 respondents 204 (84.3%) respondents have workmate who leaves the organization. For their departure from the organization they point out to the problem of low salary 113 (46.7%) and lack of opportunity for further education 54 (22.3%). Regarding the awareness of study participant regarding where the leavers are working now 94 (38.8%) of them are in the same profession (nursing practice or teaching) and most of them are working different profession like in NGO or others 109 (45.0) (Figure 9).

From 242 respondents regarding their intention to leave the current work in the coming one year and their alertness on looking other alternative job, 121 (50%) of them responded their readiness to leave the organization. From those reported to leave the organization 105 (61.8%) were looking for a job in the same profession while 52 (30.6%) were looking for a job in another profession.

### 5.2. Association between Independent and Dependent Variables (Figure 1)

As we see from the table the job satisfaction dimension shows significant correlation with each other (0.19–0.51) and was correlated with the total (overall) satisfaction score (0.43–0.66).

Intention to leave the organization was significantly associated with marital status (*P* < 0.05), whether nurses were assigned to health center or hospital (*P* < 0.05), autonomy (*P* < 0.05), working environment and group cohesion (*P* < 0.05), and by the level of professional training available in the institutions (*P* < 0.05).

Intention to leave the organization was not significantly associated with variables like age, sex, educational qualification, working experience, and benefits and salary, while there was significant association with employment opportunity (*P* < 0.05); overall nurse satisfaction with their work (*P* < 0.05), and leadership relationships in the organizations.

Intention to leave the institutions is generally has significant association with overall satisfaction of nurses on their current work (*P* < 0.05).

### 5.3. Predictors of Nurse Job Satisfaction

In multivariable logistic regression of sociodemographic variables, working experience of staff, age, institutions, sex, and working unit of nurses were the significant predictors of overall satisfaction of workers (Table 10). This model of parameter estimate scored the Hosmer and Lemeshow test of model fit [*χ* ^2^ = 12.25,   DF = 8, *P* = 0.13] with prediction capacity of 66.5%. Accordingly those workers having work experience of 2 year to 5 years were 75% less likely to be satisfied with overall job context (AOR: 0.25 [95% CI: 0.09, 0.67]), (*P* < 0.05), compared to those having work experience of 6 months to one year. Those served 5–10 years in the profession have 78% less likely to be satisfied with overall aspects of the job (AOR: 0.22 [95% CI: 0.06, 0.75]) (*P* < 0.05) compared to those served for 6 months to one year. Another one from working unit said that those assigned at maternity unit are 7 times more likely to be satisfied with overall aspects of work compared to those working in medical unit (AOR: 7.0 [95% CI 2.05–24.05]), (*P* < 0.05). Actually, all are satisfied more than medical ward even though the prediction was not significant.

Females were less likely to be satisfied with their work by 47.4% compared to those respective male nurses (AOR: 0.53 [95% CI: 0.28, 0.99]) (*P* < 0.05).

Those having an age of 31–40 years are satisfied with work threefold more compared to age categories of 20–30 years (AOR: 3.51 [95% CI: 1.05,11.73]), (*P* < 0.05). By far the age group of 41–50 years is also 15 times more likely to be satisfied than that of 20–30 years (AOR: 15.133 [95% CI: 1.971, 116.160]), (*P* < 0.05).

**Figure 2:**
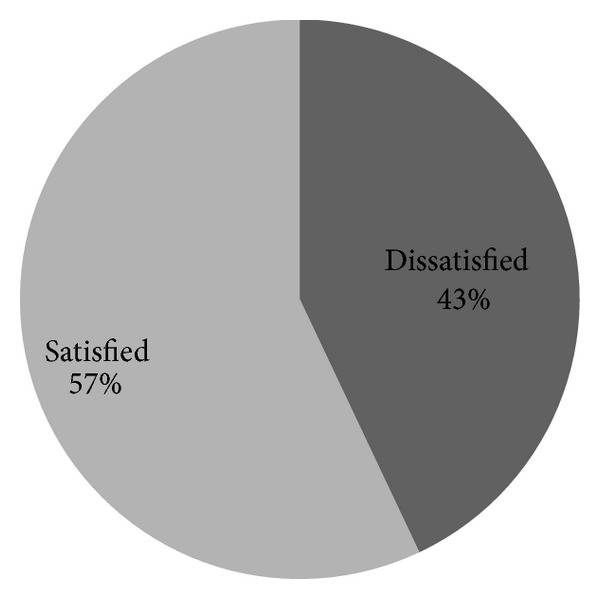
Satisfaction of nurse on leadership relationship, Sidama zone public health institutions, May-June 2010.

**Figure 3:**
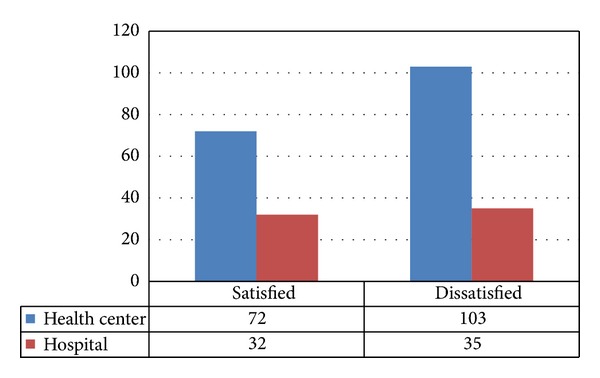
Percentage of nurses satisfied and dissatisfied with level of promotion in the working institutions, Sidama zone public health facilities, May-June 2010.

**Figure 4:**
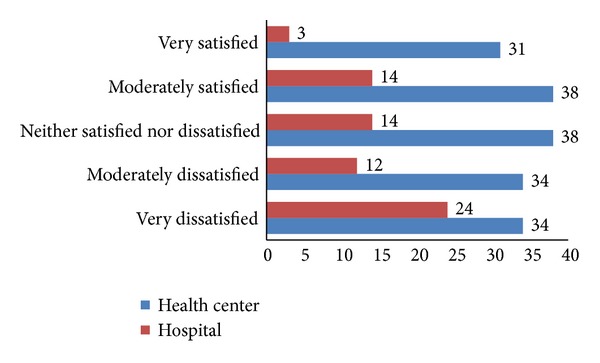
Level of nurse satisfaction on stimulating action of the working environment, Sidama zone public health facilities, May-June 2010.

**Figure 5:**
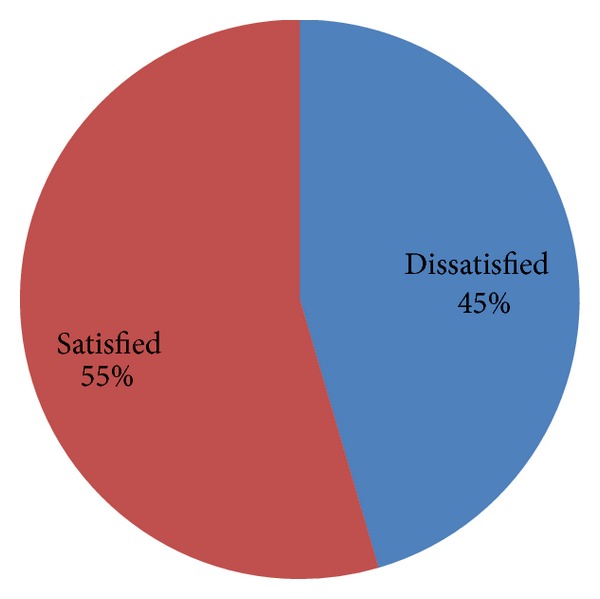
Pie overall satisfaction of study participant on working environment and group cohesion, Sidama zone public health facilities, May-June 2010.

**Figure 6:**
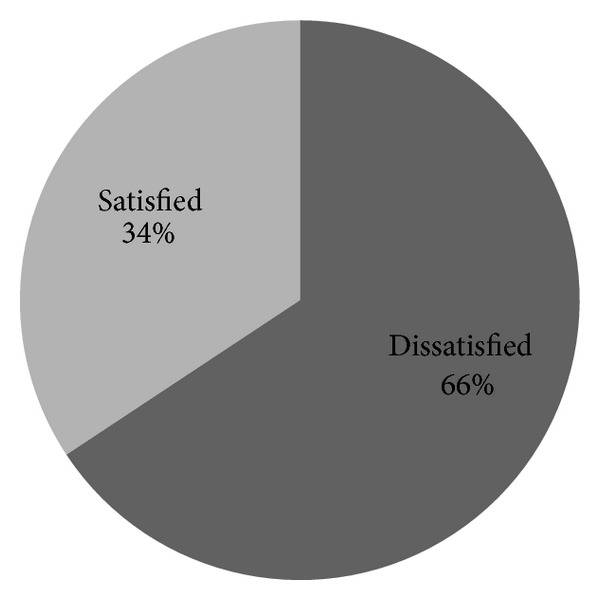
Distribution of satisfaction level of benefits and pay subscale among Sidama zone public health facilities, May-June 2010.

In binary logistic regression, statically significant predictors were identified from job satisfaction subscale for fitting overall job satisfaction model (Table 12). Accordingly, the first three most powerful predictors of overall job satisfaction are those satisfied with leadership relationship in the organization (COR: 25.56 [95% CI: 12.77–51.15]) (*P* < 0.05), those satisfied with level of autonomy at work (COR: 23.57 [95% CI: 11.87–46.80]) (*P* < 0.05), and those satisfied with training subscale (COR: 18.96 [95% CI: 9.59–37.47]) (*P* < 0.05), provided that the effects of other variables were not in the model of predictions (Figure 2).

To see the effect of job satisfaction subscale (covariate) on overall satisfaction of nurse's multivariable regression model was fitted. The model got higher prediction capacity (95.5%) with Negelkerke *R* square of 0.908 which means 90.8% of outcome variable was explained by this model and Hosmer and Lemeshow test of *χ* ^2^ = 6.12, DF = 8, and *P* = 0.63. Also, have 94.4% sensitivity with 96.5% specificity. From all job satisfaction subscale only benefits and salary were insignificant predictors of overall workers job satisfaction (AOR: 3.61 [95% CI: 0.68–18.97]) *P* = 0.129 and were rejected in the second step of backward regression method.

Those satisfied with perceived employment opportunity in the market were 12 times more likely to have satisfaction with their current job (AOR: 11.88 [95% CI: 2.64–53.49]), (*P* < 0.05). Similarly those satisfied with working environment and group cohesion at work were 27 times more likely to be satisfied with overall aspects of their job (AOR: 26.63 [95% CI: 4.26–166.19]), (*P* < 0.05) (see Table 11).

### 5.4. Predictors of Intention to Leave the Organizations

Socio demography as predictors of intention to leave the organization, all variables were fitted to the model and the model with Hosmer and Lemeshow test of model fit (*χ* ^2^ = 9.39, DF = 8, *P* = 0.31) with overall 67.4% prediction ability. While different covariates were controlled, the only significant predictors of intention to leave were marital status and working institutions. As shown below those who are single are 2.6 times more likely to leave the organization compared to those who are married (AOR: 2.56 [95% CI: 1.27–5.13]), (*P* < 0.05). Nurses who have been working in hospital are 2.49 times more likely to have intention to leave their work than their colleagues working in the health center (AOR: 2.19 [95% CI: 1.12, 4.30]), (*P* < 0.05). Actually, this finding is supported by finding from in-depth interview.

One health center head said “*…Everybody looks after good incentive but turnover tends to be higher among single than those married because those single had no nothing to worry about because they are single…*”

Nevertheless, consistently higher turnover intentions which were reported from in-depth interview were among male, lower age, and those working in rural area.

All job satisfaction subscale variables those significant in bivariate logistic regression were fitted to stepwise multivariate logistic analysis. Unfortunately only working environment and group cohesion subscale was significant predictor of worker intention to leave their current work. In fact this model scored Negelkerke *R* square of 0.19 and Hosmer and Lemeshow test *χ* ^2^ = 29.16, DF = 8, (*P* < 0.05) with 72.7% specificity with 64.46% of sensitivity on prediction. As shown in Table 13, workers who are satisfied with current work were 75% less likely to leave their current work (OR: 0.25 [95% CI: 0.12–0.51]), (*P* < 0.05). From all job satisfaction subscale, only promotion subscale was not a significant predictor in bivariate analysis, thus excluded from the model.

As stated above workers who are satisfied with overall aspect of their work were 70% less likely to have intention to leave their current working organizations provided that the effect of other factors that can influence nurse intention to turnover were not in the model. Those satisfied with working environment and group cohesion were 75% less likely to leave their current organization.

Finally job satisfaction of nurse was significantly predicted by Leadership relationship, Promotion, Autonomy, working environment and group cohesion, training, recognition at work, and employment opportunity. Working environment and group cohesion subscale were the only significant predictors of intentions to turnover.

## 6. Discussion

### 6.1. Discussion

This study aims at examining the factors influencing nurse job satisfaction and their intention to leave the organization. This is important because organization need qualified nurse and want to understand how to retain and develop competent staff compositions. Nurse job satisfaction and intention to turnover were predicted with many job satisfaction subscales while intention to leave the organizations was only predicted by working environment and group cohesion.

**Figure 7:**
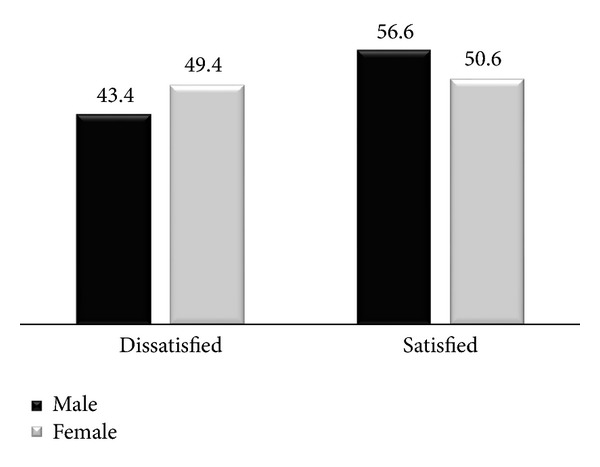
Bar distribution of overall job satisfaction of nurses in Sidama zone public health facilities, May-June 2010.

**Figure 8:**
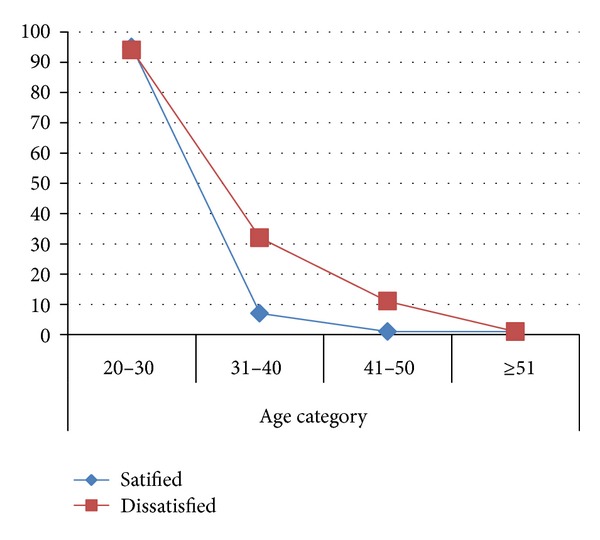
Distribution of staff overall satisfaction with respect to their age, Sidama Zone public health facilities, May-June 2010.

**Figure 9:**
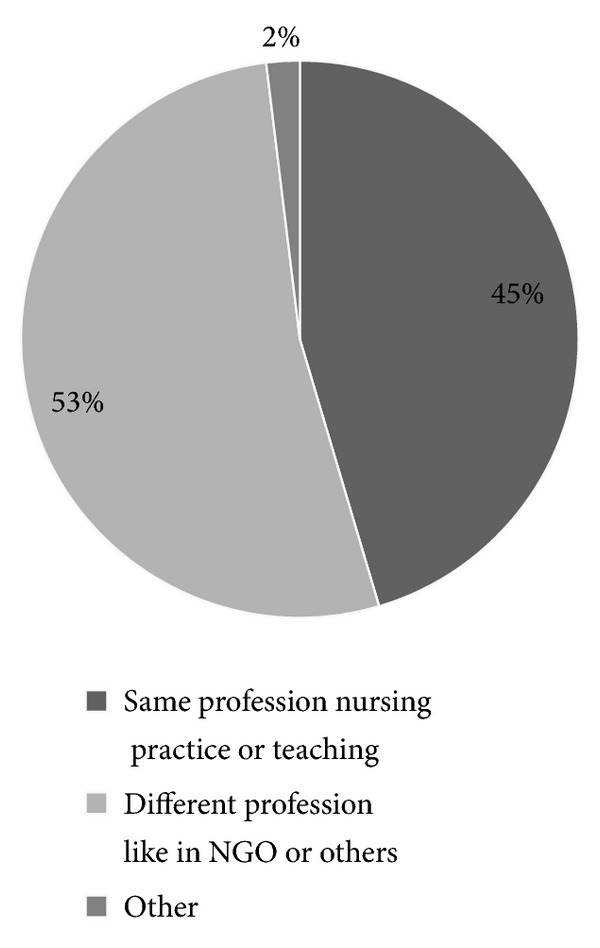
Where the departed nurses from the institutions were currently working Sidama zone public health facilities May-June 2010.

The mean age of male was 27.67 ± 4.98 and that of female was 28.35 ± 6.79, which implies that the population of nurse working in Sidama zone are young. Regarding the effect of age on job satisfaction and intention to leave the data with qualitative and quantitative study part was not held up one another. In this study age of staff nurses was significantly correlated with overall job satisfaction positively (Figure 7) (*P* < 0.05; *r* = 0.18) which is supported by findings from Greece army hospital “*Increase in job satisfaction of Army RNs was predicted by older nurses P* = 0.001, *r* = 0.363*, by more experienced ones with more years at work P* = 0,004, *r* = 0.326 [47]. But in other directions results contradict with the study of Stone et al. and of the study done in nurses of China hospitals, which says “*younger nurses with less work experience, lower professional titles *…*experience lower levels of emotional exhaustions, which is positively related with job satisfaction and negatively with intention to turnover* [25, 29].” Actually one health center head said *“… Those newly graduated nurse were highly motivated for working and they have higher satisfaction with their jobs…” *In terms of intent to leave the organization, age correlated insignificantly with intention to leave the organization while their correlation was negative. Another literature review of nurse turnover concludes that “… there is an inverse relationship between age and turnover …” [45].

Nursing is one of numerous professions in which one sex comprises the clear majority of workers while gender difference reported as factor for job satisfaction and nurse turnover [30]. In a study done on nurses working at four army hospitals and one university hospital in Northern Greece in 2007, they found that *men civilian RNs were significantly more likely to be very satisfied *(*P* = 0.037) [47], while this study also supports these results as female nurses are 47.4% less likely to be satisfied with their current job aspect. Even though sex is significantly able to predict overall job satisfaction, there is no finding showing significance difference between male and female on intention to leave the organization as it is also supported by the work of Rambur et al. [19].

Again this study identifies that those workers with work experience of 2–5 years was 75% less likely to be satisfied with overall job context (OR: 0.25 [95% CI: 0.09, 0.67]), (*P* < 0.05), compared to those having work experience of 6 months to one year. Those having work experience of 5 years to less than 10 years were 78% less likely to be satisfied with overall aspect of job (OR: 0.24 [95% CI: 0.06–0.96]) (*P* < 0.05) compared to those having work experience of 6 months to one year. In contrary to this in a study done on community hospital nurses found that RNs with more years of work experience reported higher job satisfaction, lowest levels of burnout, and were less likely to leave their positions [26]. This difference may be because of difference in sociodemographic difference in study area and it indicates further investigation of the relationship of job satisfaction and intention to leave the organization with work experience.

As one informant of in-depth interview said “*… as years of experience goes on the chance of attaining marriage increases and as the same time they will tied with social relationship and they tend to stay there….*” The quantitative data analysis shows insignificant association of work experience with job satisfaction and intention for turnover. In a study done in Macao, nurses with less than five years' work experience were 4.62 times more likely to leave than nurses with 10+ years' (*P* < 0.05) work experience [13].

Even though it was not explained well there is controversial result found from this study regarding job experience and age of worker. In one direction the level of satisfaction increases as age of nurses increases, while the overall satisfaction decreases as their level of expertise or experience increases in other directions. This issue needs further investigations and explanations.

A conscious decision should be taken to put in place leadership programmes for nurses including mentorship and coaching programmes, succession planning, carefully planned deployments to increase exposure to diverse leadership environments, recognition, and reward for expertise and excellence [10]. A leader's behavior or leadership style may influence the subordinates' level of job satisfaction. Studies have been carried out to determine how leadership behaviors can be used to influence employees for better organizational outcome. Many studies concluded that effective leadership is associated with better and more ethical performance [13]. Leadership that values staff contribution promotes retention, evidenced by consistent themes in the literature relating to autonomy, good working relationships, and a management style that facilitates rather than directs [45]. From the study participants 57% reported that they were satisfied with current style of leadership. Those satisfied with leadership were 23 times more likely to be satisfied with overall job aspect (AOR: 23.30 [95% CI 5.017–108.215]) (*P* < 0.05) and 29% less likely to leave their current organization (AOR: 0.710 [95% CI: 0.352–1.433]) *P* = 0.34. Leadership relation is able to predict overall satisfaction than intention to turnover. Leadership relationships were significantly, strongly, and positively correlated (*n* = 242), *r* (242) = 0.66, (*P* < 0.05), with overall satisfaction of nurse. This result is supported by research done on Jordanian nurse and they found positive correlation of (*n* = 55), *r* (55) = 0.91, *P* < 0.05, between transformational leadership and job satisfaction and “…the predictor, transformational leadership accounted for an estimated 80 percent of the variance of job satisfaction” [48]. The slight difference in correlation power may be because of the difference in socio demographic difference between two study areas, which probably affects their perception of leadership relationship.

Job satisfaction of worker is good in organization autonomy in the area of practice were kept [49]. In Greece, one study found the lack of significant influences of autonomy and professional growth might be identified as “dissatisfies” [47]. Most of worker were dissatisfied 128 (52.9%) with general autonomy level in their institutions while we found strong positive correlation (*n* = 242), *r* (242) = 0.65, (*P* < 0.05), with overall job satisfaction and negative correlation (*n* = 242), *r* (242) = −0.23, (*P* < 0.05), with intention to leave the organization.

Nursing education and training play an important role in the production of well-trained and properly groomed nurses. Career development and life-long learning activities in nursing promote job satisfaction, increased retention of nurses, and enable continued provision of high-quality care [33]. In this study the mean score for promotion subscale is (2 ± 0.8) in which only 100 (41.3%) were satisfied and the rest 142 (58.7%) scored dissatisfaction with promotion available in their respective institutions. This result is comparable with findings of South African hospital nurses which was (2.59) for their career development opportunities.

Lack of career advancement opportunities and unsupportive hospital policies and practices on Palestinian nurse contributed to their job dissatisfaction [34]. Those satisfied with promotion were 16 times more likely to be satisfied with their job (AOR 16.28 [95% CI: 2.71–97.6]) (*P* < 0.05) than those dissatisfied. In addition, satisfaction with promotion was the only work related variable to make a significant contribution to the prediction of turnover intention for RNs [32]. However, promotion subscale is not a significant predictor of intention to leave the organization (AOR: 1.39 [95% CI: 0.73–2.64]) *P* = 0.322. Similarly, in this study those dissatisfied with training and promotion have lower level of job satisfaction and have positive correlation with intention to leave the organizations.

Job satisfaction would be higher in work environment in which supervisors and subordinates consult together and individuals are involved with peers in decision making and task definition [50, 51]. In the area where practice environment that enables nurses to fulfill their expectations [50] and communication between nurse and physician are good, the level of nurse turnover was low [49, 52]. Working environment and group cohesion was the only significant predictor of intention to turnover from all job satisfaction subscale. This finding supported by the work of Joshua-Amadi (2003); it said low pay, poor work environment such as high workload and low social support, and high level of stress are among the reasons indicated by nurses for leaving nursing [53]. Those satisfied with working environment and group cohesion were 75% less likely to leave their current organization (AOR: 0.25 [95% CI: 0.12–0.51]) (*P* < 0.05) and 26. 6 times more likely to be satisfied with their job (AOR: 26.63 [95% CI: 4.26–16.20]) (*P* < 0.05). Again working environment and group cohesion were negatively correlated with intention to leave (*n* = 242), *r* (242) = −0.37, (*P* < 0.05) and positively with job satisfaction (*n* = 242), *r* (242) = 0.61, (*P* < 0.05).

Recognition of staff can be one of the easiest, cost effective strategies to retain experienced mature nursing staff [54]. One study on the identification of factors that influence nurse job satisfaction by a cross-sectional study of secondary data of South Carolina Hospital, Ma et al., found that 48% of nurses who held their job more than one year were very or somewhat dissatisfied with the recognition they receive [55]. Comparably this study also found 49.6% of dissatisfaction on their current level of recognition. The slight difference may be attributed to sampling, size, and difference in sociodemographic characteristics of two populations. Promoting and advocating for a culture that encourages recognition at workplace might help in retaining staff and thus enhance the quality of nursing care. Researcher from Jordan found that the correlations between intention to stay at work and recognition for outstanding/excellent performance and recognition for achievements were (*r* = 0.21, *P* < 0.01) and (*r* = 0.23, *P* < 0.01), respectively. That is, nurses who perceived having more recognition for their outstanding performance or their achievements reported a higher level of intention to stay at work than nurses with less recognition [41]. Likewise this study found nurses who are satisfied with recognition level of their institutions were less likely to leave their current organization (*r* = −0.19, *r* (242) *P* < 0.01) and have more satisfaction with their job (*r* = 0.59, *r*  (242) *P* < 0.05) compared to those dissatisfied with level of recognition. More powerfully those who are satisfied with recognition were 39 times more likely to be satisfied with overall aspect of job and 56% less likely to leave their current organization provided that the effect of other variable is not in the model.

Scored means for benefit and salary subscale for this study and study done in South Africa were 1.76 and 2.02, respectively [37]. In both cases, nurses were dissatisfied with this subscale. Additionally, benefit and salary subscale was the least powerful predictor of job satisfaction (COR: 9.64 [95% CI: 0.50,1.87]), (*P* < 0.05) and intention to turnover (AOR: 0.969 [95% CI: 0.50,1.867]), *P* = 0.925. Satisfaction with salary and benefit subscale was showing positive and significant correlation with overall job satisfaction (*r* = 0.46 *r* (242), (*P* < 0.05)) and negative and significant correlation with intention to turnover (*r* = −0.13 *r* (242), (*P* < 0.05)). Supplementary to this, Macaon nurses who scored as unsatisfied with pay and benefits were 4.14 times more likely to leave than nurses who scored as satisfied (*P* < 0.001) [13]. The difference may be because of difference in sociodemographic characteristics. In fitting of benefit and salary as predictor of intention to leave, the variable was rejected from the multivariable regression model in the second step by the software because of insignificance. This implies that to retain more qualified nurse increasing only salary may not be effective measure because other powerful factors are there. In contrary to this most of an qualitative study participants of in-depth interview report most of workers in health center were leaving because of their dissatisfaction with benefit and pay.

The study results support Herzberg and Maslow's theories [18] which identified recognition, achievement, the nature of the work, responsibility, and advancement as characteristics that are strong determinants of job satisfaction and burnout. Results from the survey demonstrate that although nurses are generally satisfied, discrepancies were masked between levels of satisfaction with different aspects of their work, between nurses with different biodemographic characteristics. The differences in satisfaction were identified in all aspect of the work satisfaction: recognition, promotion, work environment and group cohesion, leadership relationship, benefit and salary, perceived employment opportunity, and autonomy. The biggest dissatisfaction levels were identified in benefit and salary subscale, promotion and professional training subscale.

A slightly higher than half (52.5%) of the nurses working in Sidama zone are satisfied and half of the nurses already have a plan to leave (50%) their organizations and they are looking for alternative jobs. In South Africa intention to leave the jobs was reported as (40%) while satisfaction level for Uganda was 48.7% [40]. The slight difference may be attributed to difference in sociodemographic characteristics among countries [36]. Again, as they hold greater work experience their likelihood of dissatisfaction was increased which will cause crises for the health care delivery system.

Low job satisfaction was significantly related to intention to leave the profession and results in a higher chance of considering other employment opportunities [56, 57]. Major predictor of intent to leave was job dissatisfaction, and the major predictor of job satisfaction was psychological empowerment [15]. More relevantly identified from model those dissatisfied with their job were more likely to have intention to leave their organizations.

The focus on healthy work environments began in clinical settings with the goals of improving patient safety, enhancing the recruitment and retention of nurses, and promoting excellence in clinical practice. More importantly healthy and supportive work relationships have been shown to be related to higher nurse intention to remain employed [1]. As this study clearly shows the final predictor of intention to leave was working environment and group cohesion and those satisfied with working environment and group cohesion were 75% less likely to have intention to turnover, in china also similar findings were reported by Li et al. [43]. In Canada with qualitative descriptive study, from identified eight thematic categories reflecting factors nurses described as influencing their intentions to remain employed emerged from focus groups; the first two were relationships with coworkers, and condition of the work environment. In the same study, nurses said the nature and quality of these relationships were the most important reasons that they stayed employed, and others identified that negative or unsatisfying coworker relationships were a strong impetus for leaving their jobs [28].

### 6.2. Implications for Practice and/or Policy

As indicated above and by different literature knowing the most influential factors of job satisfaction and intention to turnover is important to strengthen the staff retention activities. As we maintain the more qualified and satisfied nurse we can gain good quality of care, great patient satisfaction, minimum job related side effect, and we also save the life.

### 6.3. Strength and Limitation of the Study

*Strength*. This study is like eye opening to our country to indicate the importance of the nurse job satisfaction and its determinant factors to the health care delivery system.

It is also able to indicate relevance of the issue with retention of nurse, as the issues are important in nursing labor management.

Use of mixed methods (both qualitative and quantitative) was one advantage.

*Limitation*. Lower understanding and sense of value for research were identified at different levels during data collection.

Because the data collection was overlapping with national election time, it was difficult to have cooperation letter from woreda health department. Because of this and three times visit we were enforced to leave two woredas from the study.

Recall bias because of the method we have used.

## 7. Conclusion and Recommendations

### 7.1. Conclusion

As this study assesses the factors influencing job satisfactions and intention to turnover and the title was not previously addressed in our country it will be a great impute for the professional growth in the country. Generally, following points were identified from this thesis.

- In study, some of qualitative and quantitative data were not supporting each other.
- Population of staff nurse of Sidama zone public health facilities were younger (mean age 28.13 (±6.27)).
- Age, institutions of work, sex, working unit, and work experience were significant predictor of job satisfaction while only institutions and marital status were significant predictors of intention to leave the organization.
- Most of staff were satisfied with leadership relationship (57.0%), work environment and group cohesion (54.5%), recognition at work (50.4%), and perceived alternative employment opportunity (58.3%).
- Most of staff nurses report promotion (41.3%), autonomy (47.1%), professional training (43.8%), and salary and benefit subscale (34.3%) as dissatisfying job aspects.
- Generally, 52.5% of nurses working in Sidama zone public health facilities were satisfied with their current jobs.
- The final predictors of overall satisfaction were autonomy, leadership relationship, promotion, working environment and group cohesion, professional training, recognition at work, and perceived employment opportunity.
- The final predictor of intention to turnover was working environment and group cohesion.
- Overall job satisfaction was positively and significantly correlated with job satisfaction subscale.
- Intention to turnover was negatively and significantly correlated with job satisfaction subscale and overall satisfaction of nurses.

If we are interested in promoting nurse retention, we need to focus on modifying aspects of the eight significant subscales affecting nurses overall job satisfaction and intention to turnover rather than on modifying nurses. Nurse retention challenges and obstacles may be less about nurses and more about the organizations in which they work. Retention may be promoted by developing and implementing strategies that manage one or more of these determinants so that nurses are more satisfied with those aspects of work.

### 7.2. Recommendations

Finally based on study findings and entire research process, we would like to recommend the bodies as follows.

*For Zonal Health Bureau and Woreda Health Office*

- As most of staff are frustrated regarding benefit and salary issue we would like to strengthen further intervention regarding the issue to further motivate the staff.
- Recording and documentations of important human resource file and accessibility of their information should be improved by maintaining periodic relay of information to zonal level.
- As most of staff were satisfied with level of recognition in their respective institutions, the zonal and woreda health office should maintain the continuity of the activity by the previously employed method.
- Some woredas face the problem of responsible bodies to run some office work while the head is out. Thus, woreda health office should further exercise the issue of delegation of activity.
- Health center head, woreda health department, and zonal health office should intervene with further advancement of working environment by further investigation and identification of aspect of environment and group leading them to leave the organization.
- The zonal health office should talk and enforce all concerned stakeholders to uphold the educational opportunity for nurses.
- As most of the study participants were holding diploma they were complaining from the absence of curriculum to continue further education; the woreda health department by integrating with relevant parties should create awareness and clear the confusion there among staff nurses.
- Try to foster the understanding and participation of staff nurses to research by encouraging them to participate in the research.

*For Staff Nurses*. Have creative eye! As most of nurses were answering that they were not participating in the research, we would like to recommend them to create team and exercise research activity because every practice is expected to be evidence based. 

*For Ethiopian Nursing Association and Ministry of Health.* During the study it was difficult to get consolidate and summarized data nursing professional in the country with available method of information searching. Thus, the Ethiopian Nursing Associations (ENA) with Ministry of Health should support and encourage large scale human resource study in order to have the exact figure of the professional in the country, to investigate the real problem of nurses in the country and deal with the issue with the concerned bodies.

Most of in-depth interview participants were not comfortable regarding educational opportunity and absence of curriculum to accept 10^+3^ program staffs; thus ministry of health in collaboration with ministry of education should deal with the issue.

## Figures and Tables

**Figure 1:**
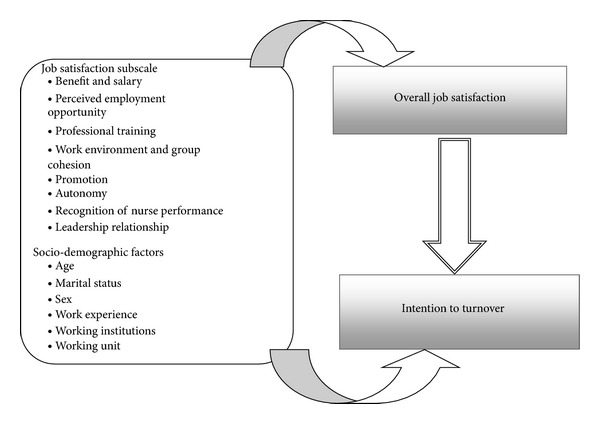
Conceptual framework showing relationship between dependent and independent variables under study.

**Table 1:** Sociodemographic characteristics of professional nurse, Sidama zone public health facility, May-June 2010.

Sociodemographic characteristics	Frequency	Percentage
Institutions		
Health centers	175	72.3
Hospitals	67	27.7
Sex		
Male	76	31.4
Female	166	68.6
Marital status		
Married	107	44.2
Single	131	54.1
Others	4	1.6
Unit of work		
Medical	28	11.6
Surgical	25	10.3
Pedi	33	13.6
OPD	65	26.9
Maternity	31	12.8
MCH	32	13.2
Others	28	11.5
Work experience		
6 month–<1 year	29	12.0
1 year–<2 years	47	19.4
2 years, <5 years	94	38.8
5 years, <10 years	37	15.3
≥10 years	35	14.5
Educational status		
BSc Nurse and midwifery	23	9.5
Diploma	219	90.5
Clinical	162	66.9
Public	22	9.1
Midwifery	25	10.3
Other	1	0.4
Age		
20–30 years	189	78.1
31–40 years	39	16.1
41–50 years	12	5.0
≥51 years	2	0.8

**Table 2:** Descriptive statistics and reliability of instruments for job satisfaction subscale, Sidama zone public health facilities, May-June 2010.

Subscales	Number of items	Mean ± SD	Alpha coefficient
Professional training subscale	4	2.50 ± 1.06	0.82
Autonomy subscale	4	3.02 ± 1.16	0.86
Work environment and cohesion	9	3.15 ± 0.93	0.86
Promotion subscale	3	2.00 ± 0.80	0.85
Benefits and salary	3	1.76 ± 0.94	0.83
Recognition at work	4	2.60 ± 1.03	0.82
Perceived alternative employment opportunity	3	2.93 ± 1.16	0.86
Leadership relationship	2	2.80 ± 1.16	0.64

**Table 3:** Frequency and percentage distribution of responses on leadership relationship, promotion, and autonomy subscale, Sidama zone public health facilities, May-June 2010.

Subscale	Satisfied frequency (%)	Neither satisfied nor dissatisfied frequency (%)	Dissatisfied frequency (%)
Leadership relationship			
(1) By the level of administration support	92 (38)	40 (16.5)	101 (45.5)
(2) By the level of recognition of your work from supervision	94 (40.1)	46 (19)	99 (40.9)

Promotion			
(1) By the level of support for continuing education	29 (12)	47 (19.4)	166 (68.6)
(2) By the level of opportunity for professional growth	16 (6.6)	52 (21.5)	174 (71.9)
(3) By the level of supporting personal growth and development through education and training	27 (11.2)	72 (29.8)	143 (59.1)

Autonomy			
(1) By level of supporting you to make autonomous nursing care decision	106 (43.8)	30 (12.4)	106 (43.8)
(2) By the level of supporting you to be fully accountable for those decisions	102 (42.1)	60 (24.8)	80 (33.1)
(3) By the chance to work alone on the job	121 (50.0)	43 (17.8)	78 (32.2)
(4) By the freedom to use your own judgment	96 (39.7)	47 (19.4)	99 (40.9)

**Table 4:** Frequency and percentage distribution of responses on work environment and group cohesion subscale, Sidama zone public health facilities, May-June 2010.

Question and subscale	Satisfied *f* (%)	Neither satisfied nor dissatisfied *f* (%)	Dissatisfied *f* (%)
Work environment and cohesion			
(1) By the level of the working environment allowing you to make autonomous nursing care decision	109 (45)	46 (19.0)	87 (36.0)
(2) By the level of the working environment allowing you to be fully accountable for those decision	112 (46.3)	51 (21.1)	79 (32.6)
(3) By the level of the working environment to encourage you to make adjustment in your nursing practice to suit patient needs	117 (48.3)	48 (19.8)	77 (31.8)
(4) By the level of the working environment to provide a stimulating intellectual environment	86 (35.5)	52 (21.5)	104 (43.0)
(5) By the level of the working environment to provide you with high level of clinical competence on your unit	108 (44.6)	44 (18.2)	90 (37.2)
(6) By the level of the working environment allowing opportunity to expand your scope of practice	105 (43.4)	40 (16.5)	97 (40.1)
(7) By the level of relationship among staff in your department	147 (60.7)	45 (18.6)	50 (20.7)
(8) By the level of group members positively influencing one another	135 (55.8)	47 (19.4)	60 (24.8)
(9) By the level of relationship with physician at your unit	110 (45.5)	51 (21.1)	81 (33.5)

**Table 5:** Frequency and percentage distribution of responses on professional training, benefit and salary, and recognition subscale, Sidama zone public health facilities, May-June 2010.

Question and subscale	Satisfied	Neither satisfied nor dissatisfied	Dissatisfied
Professional training			
(1) By the extent of training opportunities available to me	62 (25.6)	50 (20.7)	130 (53.7)
(2) By the level of training program appropriateness provided on formation to enhance nurse job performance	78 (32.2)	58 (24.0)	106 (43.8)
(3) By the level of training and orientation to new staff	80 (33.1)	41 (16.9)	121 (50.0)
(4) By the level of opportunity to participate in the research	54 (22.3)	38 (15.7)	150 (62.0)

Benefits and salary			
(1) By the level of appropriateness of your salary as compensation for your employment	26 (10.7)	20 (8.3)	196 (81.0)
(2) By the level of appropriateness for employee benefits	25 (10.3)	37 (15.3)	180 (74.4)
(3) By the level of pay in relation to what it costs to live in this area	19 (7.9)	40 (16.5)	183 (75.6)

Recognition at work			
(1) By the extent of sense of value for what you do	76 (31.4)	48 (19.8)	118 (48.8)
(2) By the level of consideration given to your personal needs	50 (20.7)	63 (26.0)	129 (53.3)
(3) By the level of consideration given to your opinion and suggestion for change in the work setting or office practice	60 (24.8)	56 (23.1)	126 (52.1)
(4) By the level of recognition of your work from peers	103 (42.6)	49 (20.2)	90 (37.2)

Perceived alternative employment opportunities			
(1) By the level of job if quite my current job and the chance that I would be able to find a job which is as good as or better than my present one.	94 (38.8)	61 (25.2)	87 (36.0)
(2) Given my age, education and the general economic condition, the chance of attaining suitable position in some other organization	90 (37.2)	60 (24.8)	92 (38.0)
(3) It would be easy to find acceptable alternative employment	80 (33.1)	58 (24.0)	104 (43.0)

**Table 6:** Satisfaction level of nurses on job satisfaction subscale, Sidama zone public health facilities, May-June 2010.

Subscale	Satisfied frequency (%)	Dissatisfied frequency (%)
Leadership relationship	138 (57.0)	104 (43.0)
Promotion	100 (41.3)	142 (58.7)
Autonomy	114 (47.1)	128 (52.9)
Work environment and cohesion	132 (54.5)	110 (45.5)
Professional training	106 (43.8)	136 (56.2)
Benefit and salary	83 (34.3)	159 (65.7)
Recognition at work	122 (50.4)	120 (49.6)
Perceived alternative employment opportunity	141 (58.3)	101 (41.7)

**Table 7:** Frequency distribution of intention to leave the organization, Sidama zone public health facilities, May-June 2010.

Variables	Frequency (%)	Percent
Do you agree to leave the organizations		
Yes	121	50.0
No	121	50.0
What kind of job are you looking for?		
*✓* Job in same profession	105	61.8
*✓* Job in another profession	52	30.6
*✓* Other	13	7.6
You have workmates (other nurses) who left your organization within the last year.		
Yes	204	84.3
No	38	15.7
Why did workmate leave the organization?		
Personal or family reasons	17	7.0
Rural nature of the working environment	16	6.6
Low salary	113	46.7
Lack of opportunity for further educations	54	22.3
Other	6	2.5

**Table 8:** Frequency distribution of intention to leave (planning) the organization, Sidama Zone public health facilities, May-June 2010.

Question	Agreement frequency (%)	Neutral frequency (%)	Disagreement frequency (%)
Do You plan to leave your organization within the next year?	143 (59.1)	73 (30.2)	26 (10.7)
Have you been actively looking for jobs in other organizations?	163 (67.4)	65 (26.9)	14 (5.8)

**Table 9:** The Pearson correlation between dependent and independent variables, Sidama zone public health facilities, May-June 2010.

Number	*N* = 242	1	2	3	4	5	6	7	8	9	10
1	Overall satisfaction	1									
2	Employment opportunity	0.49**	1								
3	Recognition at work	0.59**	0.43**	1							
4	Benefits and salary	0.46**	0.19**	0.23**	1						
5	Professional training	0.61**	0.34**	0.39**	0.31**	1					
6	Environment and group cohesion	0.61**	0.34**	0.51**	0.29**	0.29**	1				
7	Autonomy	0.65**	0.38**	0.31**	0.28**	0.45**	0.49**	1			
8	Promotion	0.43**	0.22**	0.23**	0.40**	0.31**	0.28**	0.32**	1		
9	Leadership relationship	0.66**	0.32**	0.43**	0.33**	0.48**	0.47**	0.49**	0.35**	1	
10	Intension to leave the organization	−0.29**	−0.18**	−0.19**	−0.13*	−0.22**	−0.37**	−0.23**	−0.08	−0.25**	1

**Correlation is significant at *P* < 0.01 (two tailed); *correlation is significant at *P* < 0.05 (two tailed); 1: overall satisfaction; 2: employment opportunity; 3: recognition at work; 4: benefits and salary; 5: professional training; 6: environment and group cohesion; 7: autonomy; 8: promotion; 9: leadership relationship; 10: intension to leave the organization.

**Table 10:** Parameter estimates from multivariable logistic regression model predicting overall satisfaction with respect to respondent socio-demographic variables, Sidama zone public health facilities, May-June 2010.

Predictors of overall job satisfaction	Adjusted OR (95% CI) *P* value
Age	
20–30 years	1
31–40 years	3.51 (1.05, 11.73)*
41–50 years	15.13 (1.971, 12.16)**
≥51 years	8.87 (0.000, 0)
Institutions	
Health center	2.19 (1.12, 4.3)*
Hospital	1
Sex	
Female	0.53 (0.28, 0.99)*
Male	1
Working unit	
Medical	1
Surgical	2.01 (0.59, 6.85)
Pedi	1.16 (0.44, 3.18)
OPD	3.01 (0.94, 9.61)
Maternity	7.02 (2.05, 24.06)**
MCH	1.56 (0.08, 29.41)
Chronic disease	1.79 (0.59,5.46)
Others	0.90 (0.25, 3.19)
Working experience	
6 Month–<1 year	1
1 year–<2 year	0.39 (0.14, 1.12)
2 year–<5 year	0.25 (0.09, 0.67)**
5 year–<10 year	0.22 (0.06, 0.75)*
≥10 year	0.17 (0.03, 0.82)*

*Significant at *P* < 0.05; **Significant at *P* < 0.01; CI: confidence interval.

**Table 11:** Parameter estimates from binary and multivariable logistic regression model predicting overall satisfaction with respect to job satisfaction subscale, Sidama zone Public health facility, May-June 2010.

Predictors of overall job satisfaction	Crude OR (95.0% C.I. for OR)	Adjusted OR (95.0% C.I. for OR)
Leadership relationship		
Satisfied	25.56 (12.77, 51.15)**	23.30 (5.02, 108.22)**
Dissatisfied	1	
Promotion		
Satisfied	6.28 (3.75, 12.11)**	16.28 (2.71, 97.60)**
Dissatisfied	1	
Autonomy		
Satisfied	23.57 (11.87, 46.80)**	34.35 (5.24, 225.38)**
Dissatisfied	1	
Environmental and group cohesion		
Satisfied	17.28 (9.10, 32.78)**	26.63 (4.27, 166.19)**
Dissatisfied	1	
Training		
Satisfied	18.96 (9.59, 37.47)**	36.47 (6.21, 214.37)***
Dissatisfied	1	
Benefits and salary		
Satisfied	9.64 (4.91, 18.92)**	3.67 (0.68, 18.97)
Dissatisfied	1	
Recognition at work		
Satisfied	15.66 (8.34, 29.39)**	39.18 (5.47, 279.75)**
Dissatisfied	1	
Employment opportunity		
Satisfied	8.69 (4.82, 15.69)**	11.88 (2.64, 53.49)**
Dissatisfied	1	

Significant at: ***P < 0.001, **P < 0.01, *P < 0.05.

**Table 12:** Parameter estimates from multivariable logistic regression model predicting intension to leave their current Job with respect to respondent sociodemographic variables, Sidama zone public health facilities, May-June 2010.

Predictors of intension to leave (agree to leave )	*P* value	Adjusted OR	95.0% C.I. for AOR	
			Lower	Upper
Age				
20–30 years		1		
31–40 years	0.201	2.082	0.676	6.414
41–50 years	0.157	0.166	0.014	1.992
≥51 years	0.999	3.42	0.000	.
Institutions				
Hospital	0.022	2.197	1.122	4.304
Health center		1		
Sex				
Female	0.239	1.456	0.780	2.718
Male		1		
Marital status				
Married		1		
Single	0.008	2.557	1.273	5.134
Divorced	0.808	1.446	0.074	28.353
Widowed	0.999	0.000	0.000	.
Working unit				
Medical		1		
Surgical	0.307	0.526	0.153	1.804
Pedi	0.631	0.786	0.295	2.096
OPD	0.444	1.576	0.492	5.050
Maternity	0.618	0.751	0.244	2.312
MCH	0.659	0.512	0.026	10.027
Chronic disease	0.199	0.480	0.157	1.471
Others	0.864	1.113	0.327	3.783
Working experience				
6 month–<1 year	0.658	0.712	0.158	3.207
1 year–<2 year	0.964	1.032	0.259	4.115
2 year–<5 year	0.783	1.202	0.324	4.455
5 year–<10 year	0.412	0.587	0.164	2.098
≥10 Year		1		
Educational status				
Diploma nurse		1		
Bsc nurse	0.588	1.348	0.457	3.974
Bsc midwifery nurse	0.733	1.905	0.047	77.473

CI: confidence interval.

**Table 13:** Parameter estimates from binary and multiple logistic regression models predicting intension to leave their current working organization, Sidama zone public health facilities, May-June 2010.

Predictors of intension to leave the organization	Unadjusted OR (95.0% CI)	Adjusted OR (95.0% CI)
Leadership relationship		
Satisfied	0.36 (0.21, 0.60)**	0.71 (0.35, 1.43)
Dissatisfied	1	
Autonomy		
Satisfied	0.39 (0.23, 0.65)**	0.97 (0.48, 1.95)
Dissatisfied	1	
Environmental and group cohesion		
Satisfied	0.21 (0.12, 0.37)**	0.25 (0.12, 0.51)***
Dissatisfied	1	
Training		
Satisfied	0.41 (0.24, 0.69)**	0.61 (0.31, 1.19)
Dissatisfied	1	
Benefits and salary		
Satisfied	0.57 (0.35, 0.98)*	0.97 (0.50, 1.87)
Dissatisfied	1	
Recognition at work		
Satisfied	0.44 (0.26, 0.75)**	1.22 (0.61, 2.46)
Dissatisfied	1	
Employment opportunity		
Satisfied	0.49 (0.29, 0.82)**	0.84 (0.44, 1.59)
Dissatisfied	1	
Overall satisfactions		
Satisfied	0.30 (0.17, 0.51)**	0.87 (0.29, 2.51)
Dissatisfied	1	

Significant at: ***P < 0.001, **P < 0.01, and *P < 0.05.

## List of Abbreviation and Acronyms

AGA: Agenda for Global Action
BPR: Business process reengineering
BSc: Bachelor of Science
CI: Confidence interval
DF: Degree of freedom
DWECS: Danish work environment cohort study
EU: European Union
HRH: Human resource for health
ICN: International Council of Nurses
JUSH: Jimma University Specialized Hospital
LPNs: Licensed practical nurse
LR: Logistic regression
MCH: Maternal and child health
MSc: Master of Science
OPD: Outpatient department
QWLS: Quality of work life survey (Finnish)
RN: Registered nurse
SNNPR: South Nation Nationality People Region
SPSS: Statistical package for social scientists
SQLW: Survey on quality of life in the workplace (Spanish)
SSA: Sub-Saharan Africa
JUSRH: Jimma University Specialized Referral Hospital.
